# Supplementary material for: Vitamin D Modified DSS-Induced Colitis in Mice via STING Signaling Pathway
Source: Biology (Basel). 2025 Jun 18;14(6):715. doi: 10.3390/biology14060715 (PMC12190092; doi:10.3390/biology14060715)
Supplement: Supplementary file 1 [file biology-14-00715-s001.zip › Supplementary Materials S1: 16s-report of gut microbiota/Groups/diff_analysis/RF/Class_Groups.RFimportance.pdf]

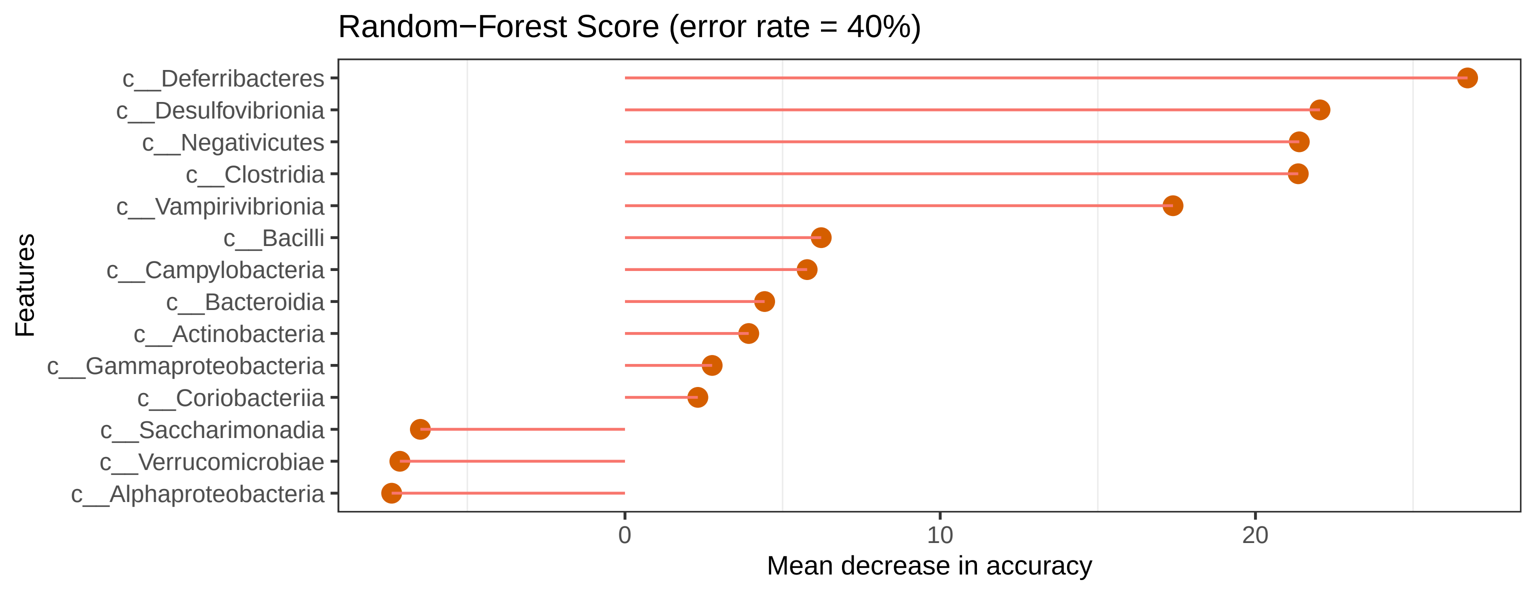
Random−Forest Score (error rate = 40%)
Features
c__Deferribacteres
c__Desulfovibrionia
c__Negativicutes
c__Clostridia
c__Vampirivibrionia
c__Bacilli
c__Campylobacteria
c__Bacteroidia
c__Actinobacteria
c__Gammaproteobacteria
c__Coriobacteriia
c__Saccharimonadia
c__Verrucomicrobiae
c__Alphaproteobacteria
0
10
20
Mean decrease in accuracy
